# Consequences of Volcanic Ash on Antioxidants, Nutrient Composition, Heavy Metal Accumulation, and Secondary Metabolites in Key Crops of Cotopaxi Province, Ecuador

**DOI:** 10.3390/toxics13020075

**Published:** 2025-01-22

**Authors:** Raluca A. Mihai, Katherine Elizabeth Rodríguez Valencia, Nina G. Sivizaca Flores, Vivanco Gonzaga Ramiro Fernando, Cubi Isuaste Nelson Santiago, Rodica D. Catana

**Affiliations:** 1 Army Scientific and Technological Research Center—CICTE, Department of Life Science and Agriculture, Universidad de Las Fuerzas Armadas—ESPE, Av. General Ruminahui s/n y, Sangolqui 171103, Ecuador; 2 Department of Life Science and Agriculture, Universidad de Las Fuerzas Armadas—ESPE, Av. General Ruminahui s/n y, Sangolqui 171103, Ecuador; kerodriguez1@espe.edu.ec (K.E.R.V.); ngsivizaca@espe.edu.ec (N.G.S.F.); rfvivanco2@espe.edu.ec (V.G.R.F.); nscubi@espe.edu.ec (C.I.N.S.); 3 Institute of Biology Bucharest of Romanian Academy, 296 Splaiul Independentei, 060031 Bucharest, Romania; rodica.catana@ibiol.ro

**Keywords:** crops, heavy metals, *Phaseolus vulgaris* L., *Zea mays* L., volcanic ash, secondary metabolites

## Abstract

This study investigates the consequences of volcanic ash on the antioxidant properties, nutrient composition, heavy metal levels, and secondary metabolites in *Phaseolus vulgaris* L. (common bean) and *Zea mays* L. (yellow corn), two crucial crops in Ecuador. The objective is to determine how volcanic ash exposure affects these crops, focusing on antioxidant properties and potential heavy metal accumulation. Field experiments were conducted in Cotopaxi Province, where both crops were cultivated under varying volcanic ash conditions. Secondary metabolites, particularly total phenols and flavonoids, were quantified using spectrophotometric methods, while heavy metal content was assessed via atomic absorption spectroscopy. Results showed a notable increase in the synthesis of secondary metabolites, especially phenols and flavonoids, in crops exposed to volcanic ash, enhancing their antioxidant capacity. Importantly, no significant heavy metal accumulation was detected, indicating that the benefits of volcanic ash application can be harnessed without associated toxicity risks. This research highlights the potential of volcanic ash to boost beneficial metabolites in yellow corn and common bean, advocating for careful agricultural practices in volcanic regions to optimize health benefits while mitigating toxicity risks.

## 1. Introduction

Throughout Ecuador’s history, agriculture and volcanic eruptions have been closely intertwined, with the agricultural sector consistently grappling with the impact and accumulation of volcanic ash on crops [1]. Cotopaxi Province (Ecuador) exemplifies this relationship, where agriculture faces significant challenges due to the continuous deposition of volcanic ash from the Cotopaxi volcano [2]. Volcanic eruptions produce ash that disperses over large areas, affecting soil and crops. Volcanic ash contains essential elements for plant biological processes, such as nickel (Ni), copper (Cu), iron (Fe), manganese (Mn), and zinc (Zn), which in the right amounts can benefit plant growth. However, it also contains non-essential, toxic elements such as arsenic (As), cadmium (Cd), lead (Pb), and mercury (Hg), which in excessive amounts can negatively impact physiological processes, becoming toxic [3]. This delicate balance between beneficial and harmful elements makes volcanic ash a crucial agricultural factor, especially in areas like Cotopaxi.

The elemental composition of volcanic ash and rocks varies considerably depending on factors such as the specific volcano’s location and the type of eruption [4]. Volcanic ash is primarily composed of dense (lithic) fragments, slag, pumice, free fractured crystals, vitreous and aggregate particles, copper oxides, and iron minerals resulting from hydrothermal alteration by hot fluids [5]. This diversity in composition affects how the ash interacts with soil and crops. The long-term impact of volcanic ash on cultivable soils becomes evident in volcanic areas where soils exhibit surprisingly high fertility upon ash settling on the surface, resulting in soils with andic properties. However, agriculture practiced on the slopes of volcanoes and adjacent valleys exposes farmers to extreme conditions during volcanic eruptions [6]. The effects of ash exposure on crops depend on characteristics, such as layer thickness, grain size, and variations in vegetation structure. Although volcanic eruptions damage crops in the short term, their development is observed in volcanic regions in the long term due to the inherent fertility of volcanic soil [7]. This phenomenon highlights the duality of volcanic eruptions as both a natural disaster and a source of renewal for agriculture.

In Ecuador, *P. vulgaris* and *Z. mays* are the key crops, being vital economically and nutritionally, occupying first place in production and consumption [8]. Common beans play a crucial nutritional role, serving as a primary source of protein, underscoring their value in the Ecuadorian diet [9]. The culture of beans is particularly important due to the rich content of bioactive phytochemicals with beneficial biological properties [10].

Maize cultivation in Cotopaxi Province is of immense importance due to maize’s pivotal role in food security, local economies, and human nutrition [1,11]. Maize is a key crop in Ecuador, significantly contributing to the agricultural economy and the cultural and dietary practices of the region. However, this crop is vulnerable to volcanic ash, which can profoundly alter its nutritional and biological properties. Investigating this impact is crucial for developing effective adaptation strategies that mitigate damage and enhance agricultural resilience in volcanic risk contexts [12,13]. Additionally, understanding the interaction between volcanic ash and maize cultivation is vital for ensuring the sustainability of agriculture in areas prone to volcanic eruptions.

Our research explores the relationship between *Phaseolus vulgaris* and *Zea mays* cultivation and volcanic ash. Both crops, pillars of the Ecuadorian diet, extend their influence far beyond borders. The impact of ash on the synthesis of secondary metabolites, which are key to human well-being and can influence potential health benefits as antioxidants, is significant. At the same time, there can be toxicity due to the absorption of heavy metals. This research is crucial for developing agricultural strategies that increase resilience and adaptation in volcanic areas. Understanding these effects will allow us to develop better agricultural practices that not only protect crops and soils but also ensure the health and well-being of the communities that depend on these crops.

## 2. Materials and Methods

### 2.1. Sample Collection

Mature fruits of *P. vulgaris* and *Z. mays* were collected from two zones: exposed (Cotopaxi Province, at 3080 m above sea level, with an ambient temperature ranging from 10 to 17 °C) and unexposed to volcanic ash (Alaques sector (0°50′37.6″ S; 78°35′15.5″ W), at an altitude of 2860 m above sea level, with temperature ranges from 15 to 20 °C).

### 2.2. Extraction of Bioactive Compounds

The extraction protocol for active compounds described by Claros [14] was employed to obtain the extracts of the two crops. Fresh and mature grains of *P. vulgaris* and *Z. mays* in the presence of volcanic ash were evaluated in two stages: Stage 1, where the soil was covered by a 3 cm layer of ash, and Stage 2, where the ash was no longer visible on the surface but was integrated into the soil due to rain. The grains were ground in a mortar to obtain flour, from which 1 g was weighed and used. Each gram of sample was macerated with 10 mL of 96% ethanol in 15 mL Falcon tubes. The mixture was manually stirred with a glass rod and then refrigerated at 5 °C for 72 h, allowing for the efficient extraction of active compounds. The assays were performed in triplicate, and the absorbance of the extracts was measured using a UV-Vis spectrophotometer.

### 2.3. Determination of Active Ingredients

In both cases, total phenolic compounds were determined using the Folin–Ciocalteu colorimetric method, following the protocol described by López-Froilán et al. [15]. For this, a small amount of water and 1.0 mL of a diluted sample (1:10) or standard solution was mixed with 1.0 mL of the Folin–Ciocalteu reagent. After 4 min, 4 mL of Na_2_CO_3_ (100 mg/L) was added, and the mixture was brought up to 25 mL with distilled water and incubated at room temperature, for 90 min, in the dark. All tests were performed in triplicate. A blank was prepared by replacing the sample with ethanol. The absorbance of the analyses was measured at a wavelength of 750 nm using a spectrophotometer. Results were calculated using a linear regression based on gallic acid (0–250 mg GAE/L) and expressed in milligrams of gallic acid per liter (mg GAE/L), yielding the equation y = 0.0061x + 0.1393 with a determination coefficient R^2^ = 0.9941.

Flavonoid content in both crops was also determined using a colorimetric method based on the formation of complexes with aluminum chloride, as described by Pekal et al. [16]. For this, 1 mL of the crude extracts was mixed with 1.5 mL of a solvent, 100 µL of CH_3_COONa (1 M), 100 µL of AlCl_3_ (10% *v*/*v*), and 2.3 mL of distilled water. The samples were allowed to rest for 40 min at room temperature, after which absorbance at 435 nm was measured. The calibration curve was prepared using quercetin in a concentration range of 0 to 1.5 mg/L, yielding the equation y = 0.0296x + 0.067 with a correlation coefficient R^2^ = 0.9878.

### 2.4. Evaluation of Antioxidant Capacity

The crops’ antioxidant capacity, both exposed and unexposed to volcanic ash, was evaluated using three different methods: ferric-reducing antioxidant power (FRAP), α-diphenyl-α-picrylhydrazyl free radical scavenging (DPPH), and free-radical-scavenging activity (ABTS).

The FRAP assay was performed following the protocol described by Agudo [17], which measures the reduction of Fe^3^⁺ to Fe^2^⁺ based on the FRAP solution prepared by mixing 100 mL of an acetate buffer (300 mM, pH 3.6), 10 mL of FeCl_3_ (20 mM), and distilled water (12 mL). For the analysis, 300 µL of the FRAP solution, 100 µL of the sample, and 300 µL of distilled water were added to each test tube, followed by a 4 min incubation in the dark, at ambient temperature. Absorbance was measured at 593 nm, and all tests were performed in triplicate, with a control prepared using ethanol instead of the sample.

The DPPH method was used to determine antioxidant activity based on the ability of antioxidants to reduce the DPPH radical, characterized by an intense purple color. Following the protocol described by Ramírez [18], 2.9 mL of the DPPH reagent and 0.1 mL of the sample were mixed, and incubated for 30 min in the dark, and absorbance was measured at 517 nm. The control was prepared similarly by replacing the sample with ethanol.

Finally, antioxidant activity was evaluated using the ABTS method, following Mendoza [19], where the ABTS^•+^ radical was generated and diluted to an absorbance of 0.76 ± 0.1 at 754 nm. To carry out the reaction, 2 mL of the ABTS^•+^ solution was mixed with 20 µL of the sample and kept for 7 min in the dark, after which the absorbance was measured at 754 nm.

### 2.5. Inductively Coupled Plasma Optical Emission Spectrometry (ICP-OES) Analysis

Inductively Coupled Plasma Optical Emission Spectrometry (ICP-OES) is a powerful analytical technique widely used for the detection of trace elements in various types of samples. The core principle of ICP-OES involves the excitation of atoms and ions in a high-temperature plasma, which is generated by coupling an inductively coupled plasma with an optical emission spectrometer. The high-temperature plasma (generated by passing an inert gas—argon—through a radiofrequency field) serves as an energetic source, atomizing and exciting the elements present in the sample, which are emitting light at characteristic wavelengths detected by a photomultiplier tube or a charge-coupled device (CCD) detector. Dry samples of *P. vulgaris* and *Z. mays* were meticulously prepared by weighing 0.5 g of finely ground plant material, which was then placed into high-resistance Teflon tubes to ensure integrity throughout the process. A carefully prepared reagent mixture, consisting of concentrated nitric acid (HNO_3_), hydrogen peroxide (H_2_O_2_), and ultrapure water, was added to each tube. The samples were subjected to a controlled digestion process utilizing a microwave digestion system (specific model and parameters referenced), tailored specifically for plant materials [20]. This method ensures the complete breakdown of the plant matrix, allowing for the accurate determination of elemental content. Post-digestion, the resultant solutions were filtered to remove any particulate matter and diluted with ultrapure water to a final volume of 10 mL in volumetric flasks. This step is critical for minimizing any potential matrix effects during the subsequent analysis. For the elemental analysis, an inductively coupled plasma optical emission spectrometer (ICP-OES), specifically the Thermo Fisher Scientific 7400 Duo model, was employed. The calibration of the instrument was achieved using a multielement ICP Mix 33 standard, with a concentration range spanning from 0.01 to 7.5 mg/L. To enhance the accuracy and precision of the measurements, yttrium (Y) was incorporated as an internal standard at a concentration of 5 ppm. This internal standard corrects any instrumental drifts and potential matrix effects, ensuring the reliability of the analytical results [21]. The analysis was conducted during the second stage of the ash presence, ensuring that all measurements were taken under consistent conditions for comparability.

### 2.6. Determination of Bioactive Compounds by LC-MS

To identify bioactive compounds in crops affected by volcanic ash in Cotopaxi Province, a high-performance liquid chromatography–mass spectrometry (HPLC-MS) approach was employed, adapting the methodology proposed by Tohma et al. [22]. The study was conducted during the ash presence, Stage 2. Ethanolic extracts were prepared from lyophilized crop samples (1 g) using 20 mL of 80% ethanol, and incubated at 30 °C for 2 h [23]. The extracts were centrifuged at 5000 rpm for 10 min at 4 °C, followed by filtration and ethanol removal via rotary evaporation at 30 °C. The resulting samples were stored in sealed plastic tubes at −20 °C until the analysis.

The HPLC-MS analysis utilized a Vanquish HPLC system (Thermo Fisher Scientific, Waltham, MA, USA) paired with an Ion Trap mass spectrometer. Chromatographic separation was achieved on an Accucore Vanquish column (150 × 2.1 mm) maintained at 35 °C with a flow rate of 0.5 mL/min [24]. A 10 µL injection volume of 0.1% formic acid served as the mobile phase. Compound identification was based on mass spectra and retention times, comparing the detected peaks with ions from standard solutions and reference databases such as PubChem, ChEBI, Metlin, and HPLC datasets. Data processing and metabolite identification were conducted using MZmine 2.53 software, supplemented with information from the scientific literature [25].

### 2.7. Statistical Analysis

The statistical analysis was performed using RStudio software (R version 4.3.2), applying a two-factor ANOVA to evaluate significant differences between groups, with a significance level set at *p* < 0.05. All experiments were conducted in triplicate, and the results were expressed as the mean ± standard deviation (SD). The correlation between secondary metabolites and antioxidant capacity was determined using a Pearson Correlation Coefficient. For ICP data visualization, representative graphs were created, focusing exclusively on metals with concentrations greater than 0.001, allowing for a clearer comparison of trace element distributions across various samples.

## 3. Results

### 3.1. Active Ingredient Determination

The total phenolic content (TPC) displayed significant differences between bean samples exposed to volcanic ash and those not exposed. Samples grown in the presence of ash exhibited higher concentrations of phenolic compounds, with an average of 0.97 ± 0.04 mg GAE/g FW, compared to 0.59 ± 0.01 mg GAE/g FW in samples without ash exposure. The same situation was observed in the case of yellow corn samples exposed to ash with a slightly higher average compared with those without ash exposure (Table 1). The total flavonoid content (TFC) showed noticeable differences between yellow corn samples exposed to volcanic ash and those not exposed (Table 1).

**Figure 1:**
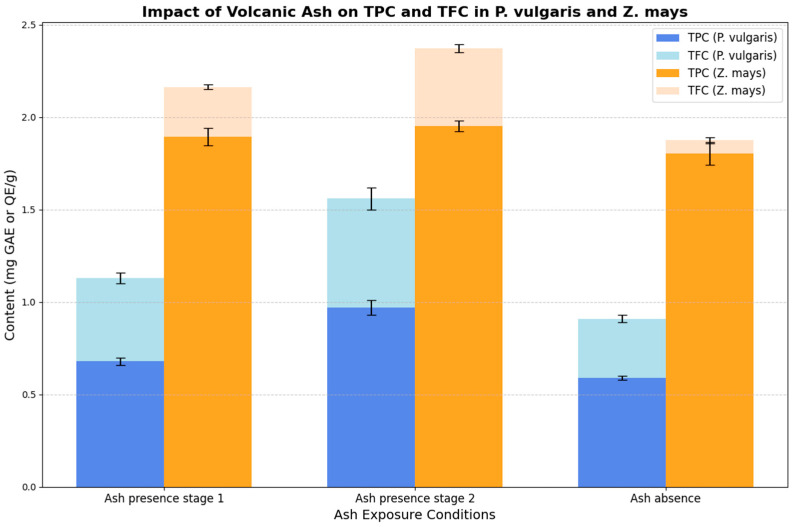
The concentration of total phenolic content (TPC) and total flavonoid content (TFC) in *P. vulgaris* and *Z. mays* under different volcanic ash exposure conditions. The data show significant differences in compound concentrations between the ash-exposed and non-exposed samples. Bars represent the mean ± standard deviation for triplicate measurements.

Figure 1 illustrates the concentration of bioactive compounds, specifically total phenolic content (TPC) and total flavonoid content (TFC), in *P. vulgaris* and *Z. mays* under different volcanic ash exposure conditions. The results show a significant increase in both compounds in crops exposed to ash, particularly in *P. vulgaris*, highlighting the positive effect of abiotic stress on the accumulation of secondary metabolites. These differences align with the literature, which associates environmental stress exposure with enhanced biosynthesis of antioxidant compounds.

### 3.2. Antioxidant Activity Determination

All three methods used for antioxidant activity determinations showed significant differences between bean samples exposed and those not exposed to volcanic ash. *P. vulgaris* samples grown in the presence of ash exhibited higher FRAP values (3.74 ± 0.34 μmol Fe^2^⁺/g FW), DPPH values (9.10 ± 0.08 μmol TROLOX/g FW), and ABTS^*+^ (8.28 ± 0.26 μmol TROLOX/g F) compared to samples grown in the zone without ash exposure (Table 2).

**Figure 2:**
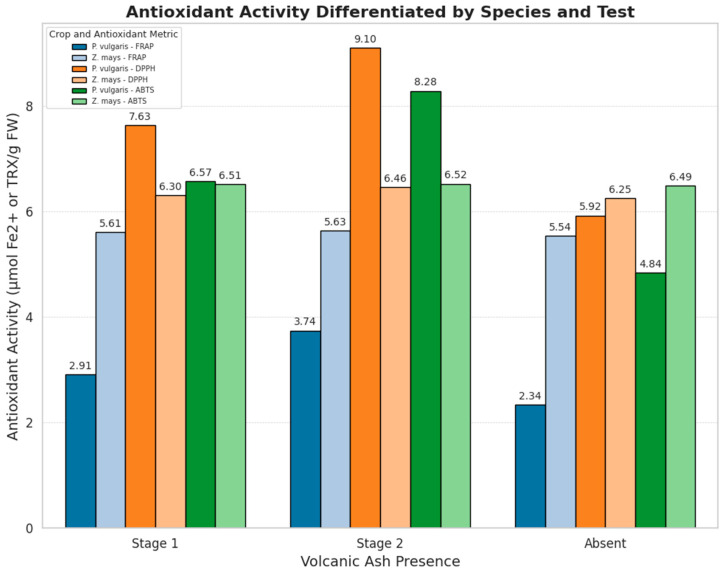
Antioxidant activity determined by FRAP, DPPH, and ABTS assays in *P. vulgaris* and *Z. mays* samples under the presence and absence of volcanic ash. The bar plot represents the mean values ± standard deviation, highlighting significant differences between conditions. The line graph illustrates trends across methods, emphasizing species-specific responses to volcanic ash exposure. Data represent triplicate measurements for each condition.

In the case of maize, antioxidant activity showed similar results for samples exposed to ash and those not exposed. Slight differences were observed between FRAP, DPPH, and ABTS radical scavenging values for both samples grown in the zone exposed to volcanic ash and unexposed (Table 2, Figure 2).

The results reveal a marked increase in antioxidant capacity in *P. vulgaris* samples exposed to ash, emphasizing the influence of abiotic stress on enhancing antioxidant potential. In contrast, *Z. mays* exhibited minimal differences between the two conditions, suggesting species-specific responses to volcanic ash exposure.

**Figure 3:**
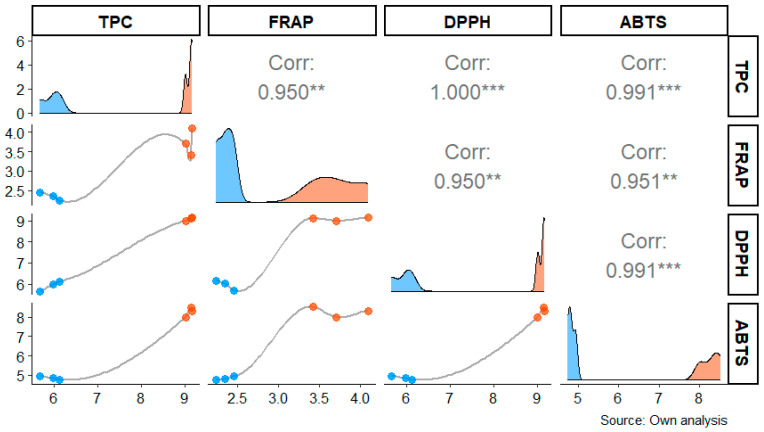
Correlation matrix between total phenolic content (TPC) and antioxidant capacity measured by FRAP, DPPH, and ABTS, in bean samples under the presence and absence of volcanic ash. Correlation coefficients (r) are indicated in each matrix cell, with statistical significance denoted by asterisks (** medium correlation, *** strong correlation). Different colors indicate data for each sample (presence and absence of volcanic ash), and the lines represent the fitted correlation trend for the respective assays. The data used include triplicate measurements for each condition.

The correlation between total phenolic content (TPC) and antioxidant capacity, measured by FRAP, DPPH, and ABTS methods, showed significant and strong relationships between the various variables in *P. vulgaris* plants in the presence and absence of ash. A very high correlation was observed between DPPH and TPC (r = 1.000 ***), as well as between ABTS and TPC (r = 0.991 ***), indicating that these antioxidant capacities are closely related in bean samples. Additionally, FRAP also showed a strong correlation with DPPH (r = 0.950 **) and with ABTS (r = 0.951 **), suggesting that phenolic compounds play a key role in the antioxidant capacity of bean plants affected by volcanic ash (Figure 3).

### 3.3. Inductively Coupled Plasma

**Figure 4:**
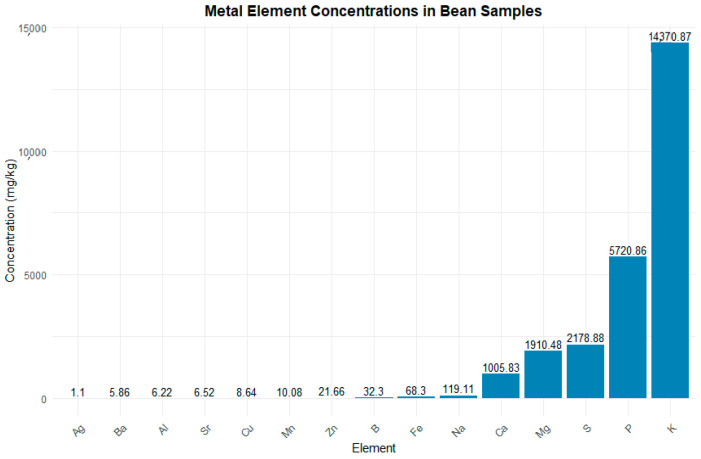
Concentrations of metallic elements in bean samples (ICP) represented in a bar graph. Legend: The graph illustrates the concentrations of various metallic elements in mg/kg found in bean samples, with potassium (K), magnesium (Mg), and calcium (Ca) being the most abundant. The *x*-axis displays the different elements, while the *y*-axis shows their respective concentrations. Metallic elements with concentrations below 0.01 mg/kg were excluded from this graph.

The ICP analysis of the bean samples exposed to volcanic ash revealed various metallic elements, only a few being present in significant concentrations. Potassium (K) had the highest concentration (14,370.87 mg/kg), followed by magnesium (Mg) with 1910.48 mg/kg and calcium (Ca) with 1005.83 mg/kg. Sodium (Na) also showed a significant concentration (119.11 mg/kg). In comparison, elements such as iron (Fe), boron (B), and aluminum (Al) had much lower concentrations, being 68.30 mg/kg, 32.30 mg/kg, and 6.22 mg/kg, respectively. Other elements, such as zinc (Zn), manganese (Mn), and copper (Cu), were present in lower concentrations, while some, like bismuth (Bi), cobalt (Co), and thallium (Tl), were detected at levels near the detection limit, around 0.01 mg/kg (Figure 4).

**Figure 5:**
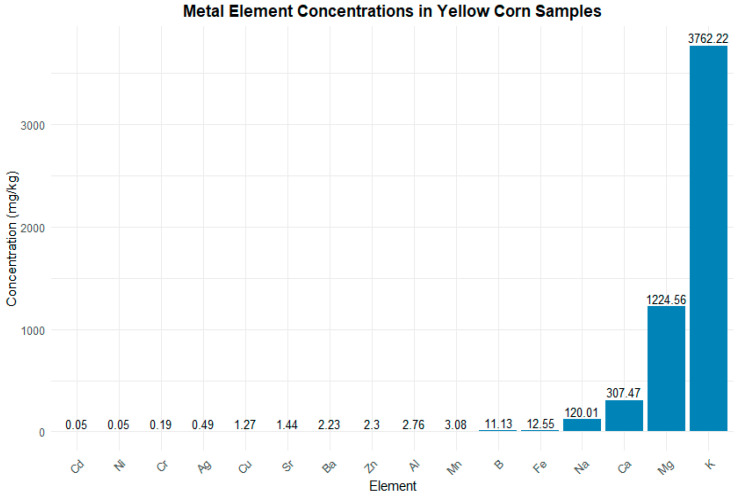
Concentrations of metallic elements in yellow corn (ICP) represented in a bar graph. Legend: The graph shows the concentrations of various metallic elements in mg/kg in yellow corn, highlighting potassium (K), magnesium (Mg), and calcium (Ca) as the most abundant elements. The *x*-axis indicates the different elements, while the *y*-axis represents the concentration.

In the case of yellow corn, the ICP analysis revealed that K stood out with the highest concentration (3762.22 mg/kg), followed by Mg (1224.56 mg/kg), Ca (307.47 mg/kg), and Na (120.01 mg/kg). Elements like Fe (12.55 mg/kg), B (11.13 mg/kg), and Al (2.76 mg/kg) showed much lower concentrations. Other elements present in low concentrations included Zn, Mn, and Cu, while elements such as Bi, Co, and Tl were detected at levels near the detection limit, around 0.01 mg/kg. The visual analysis underscores the significant differences in the concentration of metallic elements in maize, highlighting that only a few elements are present in appreciable amounts (Figure 5).

### 3.4. LC-MS Determination

The influence of volcanic ash deposition on the bioactive and metabolic composition of crops in Cotopaxi Province, Ecuador, was elucidated through the high-resolution HPLC-MS analysis. The study identified a diverse spectrum of compounds, underscoring the intricate biochemical adaptations of plants to environmental stressors. Key metabolites detected in the positive ion mode included daphnetin (C_9_H_6_O_4_), a coumarin with potent antioxidant activity (RT: 4.424 min), and argininosuccinate (C_10_H_18_N_4_O_6_), a pivotal intermediate in the urea cycle (RT: 4.898 min). Arginine (C_6_H_14_N_4_O_2_), an amino acid essential for protein synthesis and nitrogen metabolism (RT: 4.98 min), further highlighted the relevance of nitrogen-based metabolites. Similarly, the identification of pumiloside (C_21_H_24_O_11_), a glycosidic compound (RT: 5.447 min), and glycerophosphorylcholine (C_8_H_20_NO_6_P), associated with phospholipid metabolism (RT: 5.861 min), underscores the structural and functional diversity of secondary metabolites.

Phenolic and flavonoid compounds were prominently represented, with notable examples such as caffeic acid (C_9_H_8_O_4_), a hydroxycinnamic acid with dual antioxidant and anti-inflammatory properties (RT: 21.053 min), and pelargonidin-3-O-glucoside (C_21_H_21_O_10_), an anthocyanin linked to stress mitigation and potential health benefits. Complex flavonoid conjugates, including vicenin (C_27_H_30_O_15_) and isoschaftoside (C_26_H_28_O_14_), were detected, highlighting the resilience of plants through the biosynthesis of bioactive molecules. In the negative ion mode, primary metabolites such as glucose-6-phosphate (C_6_H_13_O_9_P) and sugars like sucrose (C_12_H_22_O_11_) and sorbitol (C_6_H_14_O_6_) revealed the pivotal role of carbon flux redistribution under stress conditions (Table 3).

## 4. Discussion

Cotopaxi’s stunning landscapes and volcanic soil provide an interesting backdrop for agricultural endeavors. Culturing common beans in this province faces specific environmental influences due to its proximity to the Cotopaxi volcano, exposing crops to volcanic ash. This ash primarily comprises silicon, aluminum, sulfur, and iron, with alkali and alkaline earth metal oxides such as CaO, MgO, Na_2_O, and K_2_O in smaller quantities [26].

Volcanic ash has the potential to significantly alter the balance of agricultural ecosystems by impacting vegetation structure, affecting stem and leaf shape and density, which, in turn, influences the amount of ash retained in soil and plant leaves. Ash accumulation can cause direct damage, including leaf abrasion and foliage loss, as well as indirect damage by disrupting soil water and nutrient balance. These changes may affect the organic matter decomposition and nutrient availability in the soil. Despite these negative effects, volcanic ash can also have positive impacts by enhancing carbon and nitrogen availability in the soil, benefiting plant growth [6].

In small quantities, volcanic ash can act as a beneficial fertilizer spreading essential elements like copper (Cu), iron (Fe), manganese (Mn), and zinc (Zn), which are necessary for plant biological processes, but in larger amounts, it can pose significant challenges, such as obstructing sunlight, increasing plant weight, and acidifying leaves and fruits. Currently, volcanic ash soils are most widely used for growing high-value horticultural crops, leading to the growth of the local economy [27]. Under extreme conditions, critical ash exposure can markedly alter nitrogen cycles and water–oxygen exchange in the soil, affecting long-term fertility [28]. The recovery capacity of ash-affected agricultural ecosystems largely depends on the thickness of the deposited ash layer and the adaptability of plants and soil to these changing conditions [6]. Beyond its mineral bounty, volcanic ash contains non-essential toxic elements such as arsenic (As), cadmium (Cd), lead (Pb), and mercury (Hg) that can accumulate stealthily in the biosphere [3], their silent migration echoing through ecosystems. For common bean cultivation, this dual threat manifests absorption by the plants themselves and the looming specter of transfer up the food chain.

Our results showed an increase in secondary metabolite content, respectively, phenols and flavonoids in *P. vulgaris* and *Z. mays* exposed to volcanic ash in comparison with non-exposed cultivars. Similarly, Panico et al. [29] highlighted that strawberries cultivated in the volcanic soil of the Etna mountain region showed higher total phenolic content and antioxidant activity than those cultivated in soil without ash influence.

The findings regarding increased levels of phenolic compounds and flavonoids in plants exposed to volcanic ash, such as *P. vulgaris* and *Z. mays*, can be attributed to the plants’ adaptive mechanisms to environmental stress. Volcanic ash is known to pose several abiotic stresses on soil and plant life, including nutrient imbalances, alterations in pH, and variations in water retention [30]. In response to these stresses, plants often enhance the synthesis of secondary metabolites like phenols and flavonoids, which play essential roles in defense against oxidative damage [31]. The increased quantity of phenolic compounds in *P. vulgaris* and *Z. mays* may be an adaptive response. Phenols and flavonoids are recognized for their antioxidant properties, which help neutralize reactive oxygen species (ROS) generated during stress conditions [32]. Studies suggest that when plants are exposed to stressors such as volcanic ash, the increased production of antioxidant metabolites serves as a protective mechanism against cellular damage and enhances plant resilience [33]. This accumulation of secondary metabolites not only functions as a defense mechanism but also contributes to the nutritional value of the crops. Plants with higher levels of phenolic compounds and flavonoids can offer enhanced health benefits for consumers due to their bioactive properties [33]. Thus, cultivating *P. vulgaris* and *Z. mays* in volcanic ash could provide a valuable source of dietary antioxidants that promote human health. Additionally, it is known that volcanic ash improves soil fertility and alters the nutrient profile, leading to a potentially available source of minerals that may influence plant growth and development, including secondary metabolite production [34]. Minerals such as potassium and magnesium found in volcanic soils can stimulate metabolic pathways involved in the synthesis of phenols and flavonoids [35], underlining the significance of soil composition in the biochemical responses.

Finally, the genetic makeup of *P. vulgaris* and *Z. mays* may also play a significant role in their ability to synthesize secondary metabolites in response to environmental stress [36]. Biochemical pathways leading to phenolic and flavonoid production are often regulated by specific genes, and the phenotypic differences observed between exposed and non-exposed cultivars imply a strong genetic basis for the observed accumulation of these compounds. In conclusion, the elevated levels of phenols and flavonoids in *P. vulgaris* and *Z. mays* exposed to volcanic ash underline the complex interactions between environmental stresses, plant adaptive mechanisms, and the resulting implications for human health and nutrition.

The inductively coupled plasma (ICP) analysis on both species grown in Cotopaxi Province revealed a high potassium concentration. This abundance of potassium is essential, as this element plays a crucial role in regulating water balance and in key physiological processes such as photosynthesis [37]. However, relatively low concentrations of other essential elements such as copper (Cu) and zinc (Zn) were observed. This suggests that volcanic ash may influence the availability of these nutrients in the soil, thus affecting their uptake by plants [38]. In addition, the concentrations of other essential metals, such as calcium (Ca) and magnesium (Mg), were adequate in the analyzed samples, suggesting that volcanic ash may provide essential nutrients to the soil, potentially improving its fertility [39]. However, the presence of trace metals (copper and zinc) at relatively low concentrations indicates that continuous monitoring of these elements is essential, as they are crucial for plant health and crop yield [40].

Variations in mineral composition facilitate the identification of trends that could be related to soil conditions affected by volcanic ash [41]. Trace elements such as iron (Fe) and boron (B) showed adequate concentrations, indicating that *P. vulgaris* may possess accumulation mechanisms that allow it to adapt to these conditions [42].

The ICP analysis provides crucial information on the mineral composition of both species in soils affected by volcanic ash. The findings underline the importance of assessing nutrient availability in these contexts, which is critical for developing sustainable agricultural management strategies that ensure food security and soil health [43].

The deposition of volcanic ash on crops in the Cotopaxi Province has significantly influenced the composition of secondary metabolites, antioxidants, and essential nutrients. The HPLC-MS analysis identified key bioactive compounds such as daphnetin, argininosuccinate, and ferulic acid, which play critical roles in antioxidant defense and environmental stress modulation. Recent studies highlight that daphnetin, a coumarin with potent antioxidant properties, mitigates oxidative stress and inflammation under abiotic stress conditions and in experimental models of human diseases [35,44]. This suggests that the accumulation of this metabolite in exposed crops might be a crucial adaptive response to volcanic ash impact.

Additionally, the presence of biotin, glucose-6-phosphate, and sorbitol indicates alterations in carbohydrate metabolism and essential cofactors, likely associated with increased energy and antioxidant demands due to environmental stress. The identification of phenolic acids such as caffeic acid and flavonoids like vicenin and isoschaftoside further reinforces the potential of these crops to combat oxidative stress, aligning with previous findings on their ability to protect plants against adverse conditions [45,46]. These results not only highlight the adaptive effects of Cotopaxi crops but also underscore their potential for the development of nutraceutical products and sustainable agricultural applications in challenging environments.

Heavy metals in vegetables from Ecuador have been a subject of concern, particularly due to potential contamination from agricultural practices and environmental factors. In Ecuador, the non-essential metal contamination of crops has been the focus of domestic and international studies on agricultural products. Ecuador has specific regulations, excluding all non-essential metal contaminants (harmful in certain amounts). The prolonged consumption of foods with elevated levels of heavy metals can lead to various health problems, including neurological damage, kidney dysfunction, and an increased risk of certain cancers. The specific levels of heavy metals in corn from Ecuador can vary significantly depending on the region, farming practices, and environmental conditions. Studies have shown that corn grown in Ecuador can contain detectable levels of heavy metals such as cadmium, lead [47], and arsenium [48].

In the case of corn, our analysis recorded < 0.01 mg/kg lead, Pb, which is below the CODEX STAN 193-1995 (codex norms for canned sweet corn, 1 mg/kg). In the case of the other heavy metals identified in our paper, there is no information concerning corn or beans; however, all the values identified in our samples are lower than those registered in CODEX STAN 193-1995 for different cereals and legumes.

## 5. Conclusions

Our study on the impact of volcanic ash on *P. vulgaris* and *Z. mays* in Cotopaxi Province reveals significant increases in secondary metabolites, specifically phenols, and flavonoids, in response to the environmental stresses associated with volcanic soils. These compounds not only enhance the plants’ defense against oxidative damage but also improve their nutritional value, offering potential health benefits for consumers.

The findings underscore the dual effects of volcanic ash on plants: while it contributes essential nutrients like potassium and magnesium that enhance soil fertility, it may also introduce toxic heavy metals such as arsenic and cadmium, necessitating careful monitoring of soil and crop health. In our case, these metals were not detectable. The genetic adaptability of these crops also plays a vital role in their response to environmental stress, suggesting that selective breeding could enhance resilience.

Overall, the cultivation of *P. vulgaris* and *Z. mays* in volcanic ash-affected soils presents both risks and opportunities; however, our findings demonstrate clear benefits. Understanding the implications of volcanic ash on nutrient dynamics and metabolite production can guide sustainable agricultural practices that maximize advantages while minimizing health risks, thereby supporting food production in volcanic regions. Future research should focus on long-term assessments to further explore these complex interactions.

## Figures and Tables

**Table 1:** The active ingredients in *P. vulgaris* and *Z. mays* samples exposed and not exposed to volcanic ash.

	*P. vulgaris*		*Z. mays*	
	TPC (mg GAE/g FW)	TFC (mg QE/g DW)	TPC (mg GAE/g FW)	TFC (mg QE/g DW)
Ash presence, Stage 1	0.68 ± 0.02 ^b^	0.45 ± 0.03 ^a^	1.89468 ± 0.0477 ^a^	0.26956 ± 0.0127 ^b^
Ash presence, Stage 2	0.97 ± 0.04 ^a^	0.59 ± 0.06 ^a^	1.95246 ± 0.0289 ^a^	0.41915 ± 0.0216 ^a^
Ash absence	0.59 ± 0.01 ^b^	0.32 ± 0.02 ^a^	1.80375 ± 0.0623 ^a^	0.07193 ± 0.0162 ^b^

Different letters denote significant differences.

**Table 2:** Antioxidant activity in *P. vulgaris* and *Z. mays* samples exposed and not exposed to volcanic ash.

	*P. vulgaris*			*Z. mays*		
	FRAP (μmol Fe^2+^/g FW)	DPPH (μmol TRX/g FW)	ABTS (μmol TRX/g FW)	FRAP (μmol Fe^2+^/g FW)	DPPH (μmol TRX/g FW)	ABTS (μmol TRX/g FW)
Ash presence, Stage 1	2.91 ± 0.25 ^b^	7.63 ± 0.10 ^a^	6.57 ± 0.12 ^b^	5.61 ± 0.37 ^a^	6.30 ± 0.09 ^a^	6.51 ± 0.28 ^a^
Ash presence, Stage 2	3.74 ± 0.34 ^a^	9.10 ± 0.08 ^a^	8.28 ± 0.26 ^a^	5.63 ± 0.49 ^a^	6.46 ± 0.62 ^a^	6.52 ± 0.81 ^a^
Ash absence	2.34 ± 0.11 ^b^	5.92 ± 0.24 ^b^	4.84 ± 0.10 ^b^	5.54 ± 0.45 ^a^	6.25 ± 0.06 ^a^	6.49 ± 0.31 ^a^

Different letters denote significant differences.

**Table 3:** Bioactive and Metabolic Compounds Identified in Key Crops from Cotopaxi Province Using HPLC-MS.

HPLC—MS POSITIVE IONS				
ID	Proposed Compound Identity	Molecular Formula	Retention Time	Molecular Ion
19	Daphnetin	C_9_H_6_O_4_	4.424	M + H
49	Argininosuccinate	C_10_H_18_N_4_O_6_	4.898	M+
59	Arginine	C_6_H_14_N_4_O_2_	4.98	M + H
175	Pumiloside	C_21_H_24_O_11_	5.447	M + H
218	Glycerophosphorylcholine	C_8_H_20_NO_6_P	5.861	M+
284	Biotin	C_10_H_16_N_2_O_3_S	7.111	M + H
290	L-Tyrosine	C_9_H_11_NO_3_	7.644	M + H
362	5′-Methylthioadenosine	C_11_H_15_N_5_O_3_S	15.649	M + H
407	gamma-Glutamylleucine	C_11_H_20_N_2_O_4_	16.912	M+
416	(2e)-3-(3,4-Dihydroxyphenyl)-N-[2-(4-Hydroxyphenyl)ethyl]acrylamide	C_17_H_17_NO_4_	19.959	M+
548	Glucose_6-phosphate	C_6_H_13_O_9_P	23.502	[M + Na]+
564	Sinigrin hydrate	C_10_H_16_KNO_9_S_2_	23.68	M + H
580	Isoshaftoside	C_26_H_28_O_14_	24.811	[M + H]+
605	Vicenin	C_27_H_30_O_15_	29.299	[M + Na]+
693	Abscisic acid	C_15_H_20_O_4_	31.05	M + H
859	Ferulate	C_10_H_10_O_4_	38.501	M + H
886	Pelargonidin-3-O-glucoside	C_21_H_21_O_10_	19.959	M − H
**HPLC—MS NEGATIVE IONS**				
**ID**	**Proposed Compound Identity**	**Molecular Formula**	**Retention Time**	**Molecular Ion**
62	Glucose 1-phosphate	C_6_H_13_O_9_P	5.175	M − H
70	Sorbitol	C_6_H_14_O_6_	5.192	M − H
120	Sucrose	C_12_H_22_O_11_	5.33	M − H
144	Glucose, fructose, mannose	C_6_H_12_O_6_	5.3	M − H
459	alpha, alpha-Trehalose	C_12_H_22_O_11_	19.878	M − H
503	Caffeic acid	C_9_H_8_O_4_	21.053	M + H
563	Trihydroxyflavone-C-hexoside-C-pentoside	C_20_H_20_O_11_	23.245	M − H
629	Orientin	C_21_H_20_O_11_	24.594	M − H

## Data Availability

The original contributions presented in the study are included in the article; further inquiries can be directed to the corresponding author due to privacy.
